# The Avon Longitudinal Study of Parents and Children - a resource for COVID-19 research: approaches to the identification of cases November 2020

**DOI:** 10.12688/wellcomeopenres.16808.1

**Published:** 2021-05-20

**Authors:** Kate Northstone, Mark Mummé, Ruth Mitchell, Nicholas J. Timpson

**Affiliations:** 1 Department of Population Health Sciences, Bristol Medical School, University of Bristol, Bristol, BS8 2BN, UK; 2 MRC Integrative Epidemiology Unit, Department of Population Health Sciences, University of Bristol, Bristol, BS8 2BN, UK

**Keywords:** ALSPAC, birth cohort study, COVID-19, coronavirus, online questionnaire, antibody testing, SARS-CoV-2, administrative data

## Abstract

The Avon Longitudinal Study of Parents and Children (ALSPAC) is a prospective population-based cohort study which recruited pregnant women in 1990–1992 and has followed these women, their partners (Generation 0; G0) and their offspring (Generation 1; G1) ever since. The study reacted rapidly to the coronavirus disease 2019 (COVID-19) pandemic, deploying three online questionnaires in March, May and October 2020. Home-based antibody tests accompanied the third questionnaire. In addition, linkage to Public Health England (PHE) Pillar I and II testing results has been obtained for all participants who have consented or for whom we have NHS Confidentiality approval group permitted Section 251 access.

For the purposes of ongoing study, we have identified likely cases of COVID-19 from available data. To determine likely cases, we have developed a hierarchy depending on the source of the data: self-report, antibody test result and Pillar I and II linkage and a combination thereof; providing more certainty in the case status. This data note describes how we have ascertained case status in ALSPAC. The subsequent case variable will be made available through our COVID release files alongside testing data from PHE.

## Introduction

The Avon Longitudinal Study of Parents and Children (ALSPAC) is a unique three-generational study, comprising ‘G0’: the cohort of original pregnant women, the biological father and other carers/partners; ‘G1’: the cohort of index children; and ‘G2’: the cohort of offspring of the index children. The study has a wealth of biological, genetic and phenotypic data across these generations ^1– 4^. ALSPAC has been well placed to capture information across key parts of the population in light of the coronavirus disease 2019 (COVID-19) pandemic – in particular the contrast between those in higher risk (the G0 cohort; mean age ~59 years) and lower risk (the G1 cohort; mean age ~28 years) groups. We have been able to collect repeat data quickly using our existing infrastructure for online data collection ^5, 6^. In addition, antibody tests have been undertaken alongside our last online questionnaire ^7^.

Alongside around 30 years of legacy data and biosamples, the wider COVID-19 data collection in ALSPAC includes data from three main sources: 1) self-reported data from questionnaires, 2) data from clinical services based on linkage to medical and other records and 3) information from biological samples. The data from these sources are intended to be complementary and help address different potential research questions around COVID-19. We wanted to objectively estimate how many people in the study may have been infected with the virus that causes COVID-19 and these data form the basis for ongoing and planned studies investigating recuperation ^8^ and “long COVID”. All of these downstream applications – pertinent to the investigation of COVID-19 – require an acceptable definition of cases status among the population. As a primarily observational study, our main source of data has been from self-report and it is important to determine how accurately true cases can be identified from the data provided by our participants.

This data note describes how the data collected via our online questionnaires, results from home-based antibody testing and from linkage to Public Health England (PHE) has been combined to identify likely cases of COVID-19 in ALSPAC. This data will form the basis of all subsequent analyses investigating cases of COVID-19 and will be extended as further testing results become available.

## Methods

### Setting

ALSPAC is an intergenerational longitudinal cohort that recruited pregnant women residing in Avon, UK with expected dates of delivery 1 ^st^ April 1991 to 31 ^st^ December 1992 ^1, 2^. The initial cohort consisted of 14,541 pregnancies resulting in 14,062 live births and 13,988 children who were alive at 1 year of age. From the age of seven onwards, the initial sample was bolstered with eligible cases who had originally failed to join the study and there were subsequently 14,901 children alive at 1 year of age following this further recruitment ^4^. Please note, the study website contains details of all the data that is available through a fully searchable data dictionary and variable search tool.

In response to COVID-19 it was necessary to develop a data collection strategy which was practical, would yield data quickly and could be updated and repeated if necessary. For these reasons, we chose to use an online only data collection approach for this, restricting our invites to those participants with a valid email address (and coordinated with a systematic communications/outreach campaign to obtain updated information from participants). Our questionnaires were developed and deployed using REDCap (Research Electronic Data CAPture tools ^9^); a secure web application for building and managing online data collection exercises, hosted at the University of Bristol.

### Baseline

We only included those who responded to at least one of the three COVID questionnaires and/or had a positive test via PHE.

Three sources of data are available to assist us in determining cases of COVID-19 - we describe these as follows:

### Self-report data

Online questionnaires were completed by participants in March (Q1), May (Q2) and October 2020 (Q3) ^5– 7^. The following questions were asked:

1. COVID-19 symptoms and negative control symptoms reported retrospectively, on a monthly basis, from Oct 2019 to May 2020 (Q1), May 2020 (Q2) and March 2020 to October 2020 (Q3). Yes or No responses for each symptom.
2. “Do you think that you have or have had COVID-19?” With the following options: Yes, confirmed by a positive test; Yes, suspected by a doctor but not tested; Yes, my own suspicions; No (Q1, Q2, Q3).
3. “Have you had a test before to see if you have or have had COVID-19? If yes, “What kind of test have you had?” with the following options: A swab test (swab taken from your throat or nose) which tests for active infection; An antibody test (this usually involves a drop of blood taken from your finger) which test for past infection; Other; Don’t know. If yes to any of these, have you had a positive result?” If yes, “When was the sample taken for the test that came back positive, give the latest test data if you have had more than one” (Q3).

**Figure 1.**
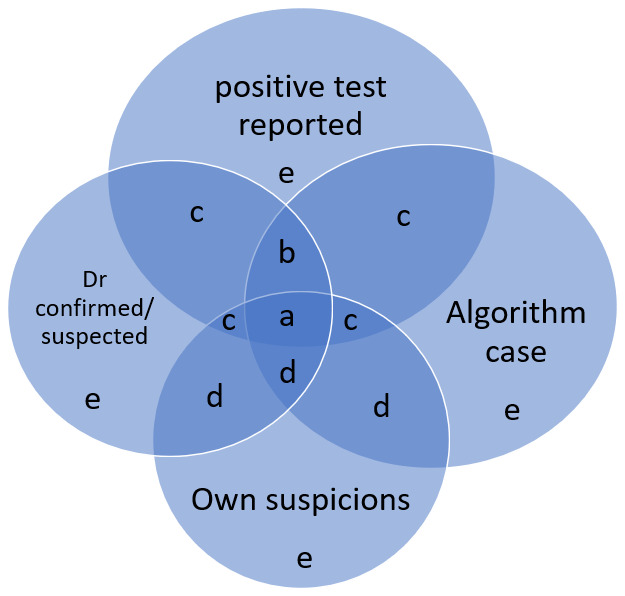
Combinations of COVID-19 case status from self-report questionnaires.

Using [1] we applied the algorithm developed by Menni *et al.* ^10^ for each set of monthly data to determine whether a participant was a ‘probable’ case in that month ^11^. Using [2] we identified those who were potentially cases as suspected by a doctor and those who self-suspected they were a case (Note, that in Q1 and Q2 very small numbers reported a positive test). Finally using [3] we identified potential cases who reported a positive test result (not from our antibody tests). We created binary variables for each of these four sources of data representing whether a participant ‘ever’ fell into one of those groups. Figure 1 shows the overlap between the four different sources of self-report data and Table 1 shows the hierarchy we have applied to this self-report data such that the higher in the table, the more likely we consider a participant is to be a true ‘case’ based on self-report data alone. Note, that we decided to drop group e from our self-report group, as we did not think this group would be reliable (see results).

**Table 1.** Hierarchy of self-report data.

	Hierarchy
a	All four sources of self-report
b	+ve test AND Dr confirmed/suspected AND predicted algorithm case
c	+ve test AND (Dr confirmed OR predicted algorithm case)
d	No +ve test but any other pair of self-report data
e	Any individual self-report only

### Serology results

Antibody tests provided by Una Health and Fortress Diagnostics Ltd. were sent to consenting participants in October 2020. The methodology is described in more detail here ^7^. Briefly, 5220 participants were sent testing kits through the post and were asked to complete an online survey as described above. We consider those who reported a positive result to IgG or IgG + IgM as reporting positively to serology.

### Linkage to PHE

Identifiers were sent to PHE via encrypted, secure file transfer for those G0 Mother and G1 participants who had consented to linking to health data (n=5201 G1 and n=1033 G0 mothers) or for whom we had permission under section 251 to request health data (G1 only, n=7573). PHE returned details of all Pillar I and II swab tests: Pillar I testing is for those with a clinical need and critical key workers delivered by PHE and NHS labs. Pillar II testing is for the wider population usually undertaken at a regional, local or mobile test unit often through commercial partnerships.

## Key results

### Self-report data

A total of 6807 participants completed Q1 (2706 original mothers, 1014 original fathers/partners, 2973 offspring ^5^), and Q2 was completed by 6482 participants (2639 original mothers, 1039 original fathers/partners, 2711 offspring and 1039 responded to Q2 but not Q1 ^6^).

In Q1, a previous positive test result was reported by eight participants, this increased to 36 in Q2 and 58 in Q3. In Q1, 77 participants reported in Q1 that their Doctor had suspicions that they had COVID-19, this increased to 91 in Q2 but went to down to 73 in Q3. A much larger number of participants suspected themselves that they had COVID-19: 865 in Q1, 838 in Q2 and 980 in Q3.

Applying the Menni algorithm to the self-reported symptom data from each questionnaire resulted in over 400 participants being a ‘probable’ case at least once in each of the questionnaires.

Table 2 summarises the responses received via self-report data and the overlap between the different question sources within the questionnaires.

**Table 2.** Responses to self-report hierarchy.

	Hierarchy	G1	G0 Mother	G0 partner	TOTAL
a	All four sources of self-report	11	4	2	17
b	+ve test AND Dr confirmed/suspected AND predicted algorithm case	21	6	3	30
c	+ve test AND (Dr confirmed OR predicted algorithm case)	17	7	1	25
d	No +ve test but any other pair of self-report data	181	80	162	423
e	Any individual self-report only	750	533	779	2062

### Serology results

In total, 4819 participants completed an antibody test. Of these,197 reported a positive response to antibodies. Of those reporting a positive result on our antibody test, 55 reported that they did not think they had had COVID-19 in Q3, emphasising the importance of not relying on symptoms and self-report only.

### Linkage to PHE

The first set of results were received in October for G1 and contained 31 positive test results, whether the test was Pillar I (n=10) or II (n=21) and the dates of the sample being provided and when the laboratory reported the test result (the difference in these dates ranged between 0 and 4 days). G0 data was also obtained later in October with nine positive results (three Pillar I and six Pillar II tests). A second set of results were received in November and provided a further 136 positive tests (11 Pillar I and 125 Pillar II) for G1 with none for G0. At the time of writing, ALSPAC has received updates from PHE to the end of January 2021 and summary data will be made available through the main resource. Future test results will be added as they become available.

### Combining all sources of data

**Figure 2.**
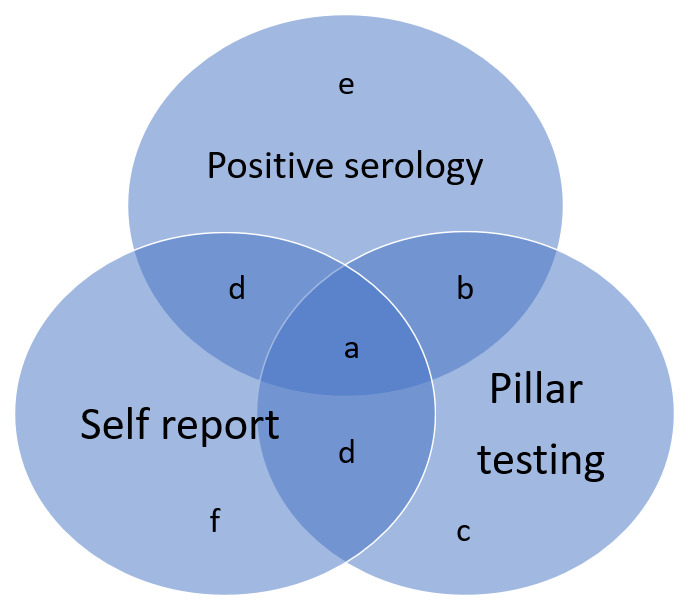
Combining all sources of data regarding COVID-19 case status.

**Figure 3.**
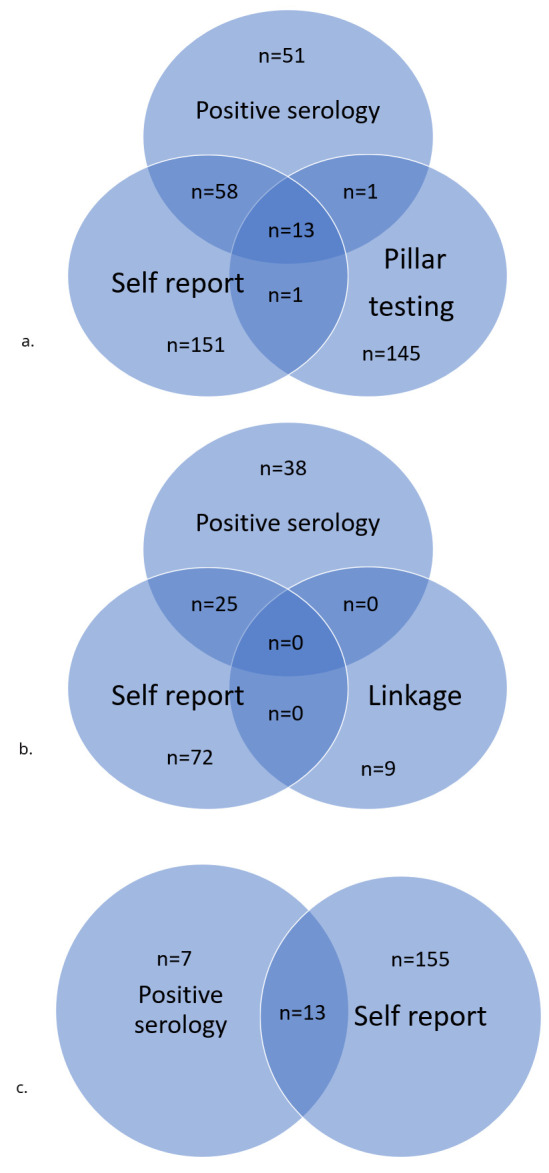
Breakdown of numbers for each participant group.

As with our self-report data we needed to consider a hierarchy for identifying cases given the multiple sources of data at our disposal. Figure 2 and Table 3 demonstrate the overlap between the data sources and the hierarchy agreed upon to best define cases ( Figure 3 and Table 4 presents the numbers falling into each category for each participant group). Pillar testing was felt to be the most reliable of the sources as it was objectively assessed and subject to strict protocols. Interestingly, there was not a great deal of overlap with pillar testing and the other sources of data. Similarly, the overlap between the antibody testing and self-report was not substantial indicating that many of the ‘cases’ identified by antibody testing were likely asymptomatic. This may partly explain the lack of overlap between antibody testing and pillar testing; individuals not experiencing symptoms will not have sought testing through the national provision. For the purposes of inviting likely cases to future research studies we have chosen to remove the self-report only group as there are sufficient numbers in the combined groups to meet that need.

**Table 3.** Hierarchy Combining all sources of data.

	Hierarchy
a	All sources
b	Pillar testing and +ve serology
c	Pillar testing with or without self-report
d	+ve serology with self-report
e	+ve serology without self-report
f	Self-report only

**Table 4.** Response to combined data hierarchy.

	Hierarchy	G1	G0 Mother	G0 partner	TOTAL
a	All sources	13	0	n/a ^a^	13
b	Pillar testing and +ve serology	1	0	n/a ^a^	1
c	Pillar testing with or without self-report	153	9	n/a ^a^	162
d	+ve serology with self-report	58	25	13	96
e	+ve serology without self-report	51	38	7	97
f	Self-report only	151	72	155	383

^a^n/a as linkage not currently available in G0 partners

## Strengths and limitations of the data

This data collection has a number of strengths: The study has been able to respond rapidly to the pandemic and collected a number of waves of data already. This has been undertaken in two generations of families. In addition, the triangulation of self-report, antibody testing and linkage to community testing is unique in a longitudinal cohort study at the time of writing.

However, there is a substantial proportion of participants who self-reported their own potential case status but did not have positive antibody or swab tests. At the time of data collection (latter end of 2020), community testing was widely undertaken in the UK. The majority of positive results available to us through PHE were from tests taken after our self-reported data collection was completed. The sensitivity of the antibody testing in the first week after symptom onset is low, meaning that we may have missed some cases who were in the early stage of infection in October. In addition, the duration of antibodies is currently unknown, meaning that we may have missed cases who were infected early in the pandemic and their antibodies have diminished ^12^. We are currently collecting our fourth sweep of self-reported data from both generations and it will be crucial to feed this data into our case descriptions as we continue to gather pillar testing information from PHE. However, it must be noted that our results are not currently comparable across generations as we do not consent for linkage in our G1 fathers, nor do we have section 251 access to the data from mothers who did not originally respond to our consent campaign. We are currently seeking permission for this and will update our case summary data accordingly. We are due to repeat home-based antibody testing in April 2021 which will further enhance our ability to assess overlap and determine the quality of self-report data.

## Consent

Ethical approval for the study was obtained from the ALSPAC Ethics and Law Committee and the Local Research Ethics Committees (Bristol and Weston Health Authority: E1808; Southmead health authority: 49/89 and Frenchay health authority: 90/8). The South Central – Berkshire Research Ethics Committee provided specific approval for this data collection. Informed consent for the use of data collected via questionnaires and clinics was obtained from participants following the recommendations of the ALSPAC Ethics and Law Committee at the time. Study participants have the right to withdraw their consent for elements of the study or from the study entirely at any time. Full details of the ALSPAC consent procedures are available on the study website ^13^. Participants consented electronically to take part in the antibody testing. Approval for PHE linkage was obtained from CAG (REF: ECC 1–05(b)/2012).

## Data availability

### Underlying data

ALSPAC data access is through a system of managed open access. The steps below highlight how to apply for access to the data included in this data note and all other ALSPAC data:

1. Please read the ALSPAC access policy ^14^ which describes the process of accessing the data and samples in detail, and outlines the costs associated with doing so.

2. You may also find it useful to browse our fully searchable research proposals database ^15^, which lists all research projects that have been approved since April 2011.

3. Please submit your research proposal ^16^ for consideration by the ALSPAC Executive Committee. You will receive a response within 10 working days to advise you whether your proposal has been approved.

Please note that a standard COVID-19 dataset will be made available at no charge (see description below); however, costs for required paperwork and any bespoke datasets required with additional variables will apply. Access to data from Public Health England is restricted to study staff and thus only the derived ‘case’ status variables can be onward shared.

Case status data is available in two ways:

1. A freely available standard set of data containing *all* participants together with key sociodemographic variables (where available) is available on request (see above). This dataset includes data obtained from the first three COVID questionnaires. Subject to the relevant paperwork being completed (costs may apply to cover administration) this dataset will be made freely available to any bona fide researcher requesting it. A full list of variables released is available here: https://doi.org/10.17605/OSF.IO/6JR7E.
2. Formal release files have been created for G0 mothers, G0 fathers and G1 participants in the usual way and now form part of the ALSPAC resource (due to the small number of G1 partners contributing we will not be formally releasing this data, however, it may be available on request for specific G2 projects). These datasets (or sections therein) can be requested in the usual way. Frequencies for all variables for each participant group are available in the data dictionary in the usual way ^17^.

### Extended data

Open Science Framework: ALSPAC COVID-19 Questionnaires. https://doi.org/10.17605/OSF.IO/Q46R7 ^11^.

This project contains the following extended data:

1. deriving case status_ALSPACYPs_Nov2020.do (The Stata do file used to create case status).
2. ALSPAC_COVID_Pillartesting_varlist.pdf (List of variable names and labels for all COVID data collection including PHE testing data).
3. PHE_CVOID_YP_1a.pdf (Associated data dictionary including frequencies of all COVID variables that are available).

Data are available under the terms of the Creative Commons Attribution 4.0 International license (CC-BY 4.0).
