# Supplementary material for: The Avon Longitudinal Study of Parents and Children - a resource for COVID-19 research: approaches to the identification of cases November 2020
Source: Wellcome Open Res. 2021 May 20;6:122. [Version 1] doi: 10.12688/wellcomeopenres.16808.1 (PMC11109715; doi:10.12688/wellcomeopenres.16808.1)
Supplement: Peer review history

# Reviewer report: Reviewer response for version 1

**DOI:** 10.21956/wellcomeopenres.18541.r70263

**Published:** 2024-05-11

**Authors:** Eric J Nilles

**Affiliations:** 1 Brigham and Women’s Hospital, Boston, MA, USA

The authors describe the Avon Longitudinal Study of Parents and Children which enrolled pregnant women in 1990-1991 and followed the women, their partners and their children since. The current data note specifically describes elements of the study implemented in response to the COVID-19 pandemic that includes three rounds of questionnaires and home-based antibody testing and linkage to PHE pillar I and II. The methods including determination of infection status are rigorous and well described and overall this study could provide valuable and granular data for researchers on a large cohort early in the pandemic.

Are sufficient details of methods and materials provided to allow replication by others?

Yes

Is the rationale for creating the dataset(s) clearly described?

Yes

Are the datasets clearly presented in a useable and accessible format?

Yes

Are the protocols appropriate and is the work technically sound?

Yes

Reviewer Expertise:

Infectious disease epidemiology, immunoepidemiology, serological surveys and population-based field studies

I confirm that I have read this submission and believe that I have an appropriate level of expertise to confirm that it is of an acceptable scientific standard.

# Reviewer report: Reviewer response for version 1

**DOI:** 10.21956/wellcomeopenres.18541.r44013

**Published:** 2021-06-09

**Authors:** Gillian Santorelli

**Affiliations:** 1 Bradford Institute for Health Research, Bradford, UK

This data note describes a hierarchical approach to identifying COVID-19 infections using self-report, antibody test result, Pillar I and II linkage, and a combination of the above. The methodology is described clearly, and the paper offers guidance on how best to ascertain case status. The extended data available online provides further information on the data collected, response rates and syntax on how case status was derived. This is a well written and informative note that will be a valuable resource to fellow researchers.

Are sufficient details of methods and materials provided to allow replication by others?

Yes

Is the rationale for creating the dataset(s) clearly described?

Yes

Are the datasets clearly presented in a useable and accessible format?

Yes

Are the protocols appropriate and is the work technically sound?

Yes

Reviewer Expertise:

Epidemiology of chronic and acute morbidity.

I confirm that I have read this submission and believe that I have an appropriate level of expertise to confirm that it is of an acceptable scientific standard.

# Response

**Published:** 2021-06-10

**Authors:** Kate Northstone

**Affiliations:** University of Bristol, UK

We thank the reviewer for their positive comments.
